# Non contiguous-finished genome sequence and description of *Dielma fastidiosa* gen. nov., sp. nov., a new member of the Family *Erysipelotrichaceae*

**DOI:** 10.4056/sigs.3567059

**Published:** 2013-06-13

**Authors:** Dhamodharan Ramasamy, Jean-Christophe Lagier, Thi Tien Nguyen, Didier Raoult, Pierre-Edouard Fournier

**Affiliations:** 1 Aix-Marseille Université, URMITE, Faculté de médecine, Marseille, France

**Keywords:** *Dielma fastidiosa*, Genome, Culturomics, Taxono-genomics

## Abstract

*Dielma fastidiosa* strain JC13^T^ gen. nov., sp. nov. is the type strain of *D. fastidiosa* gen. nov., sp. nov., the type species of a new genus within the family *Erysipelotrichaceae*. This strain, whose draft genome is described here, was isolated from the fecal flora of a healthy 16-year-old male Senegalese volunteer. *D. fastidiosa* is a Gram-negative anaerobic rod. Here we describe the features of this organism, together with the complete genome sequence and annotation. The 3,574,031 bp long genome comprises a 3,556,241-bp chromosome and a 17,790-bp plasmid. The chromosome contains 3,441 protein-coding and 50 RNA genes, including 3 rRNA genes, whereas the plasmid contains 17 protein-coding genes.

## Introduction

*Dielma fastidiosa* strain JC13^T^ (CSUR P149 / DSM 26099) is the type strain of *D. fastidiosa* gen. nov., sp. nov., the type species of *Dielma* gen. nov. This bacterium is a Gram-negative, anaerobic, catalase and indole-negative bacillus, isolated from the stool of a healthy Senegalese patient as part of a study aimed at cultivating individually all species within human feces [1,2]. The conventional genotypic methods used in bacterial taxonomy include 16S rRNA gene-based phylogeny and nucleotide similarity [3,4], determination of the G + C content and DNA–DNA hybridization (DDH) [5,6]. Although DDH and 16S rRNA gene similarity cutoffs are considered as gold standards in bacterial taxonomy, they have some limitations as they do not apply well to all species or genera [3]. Hence, there is a need for alternative methods. The introduction of high-throughput genome sequencing and proteomic analyses [7] provided a source of comprehensive information about studied bacterial isolates. Such data may now be included among the criteria used for taxonomic identification. We recently proposed to use a polyphasic approach to describe new bacterial taxa that is based on their genome sequence, MALDI-TOF spectrum and main phenotypic characteristics [8-26].

Here we present a summary classification and a set of features for *D. fastidiosa* gen. nov., sp. nov. strain JC13^T^ (CSUR P149 / DSM 26099) together with the description of the complete genome sequencing and annotation. These characteristics support the circumscription of the genus *Dielma* and its type species, *D. fastidiosa* within the family *Erysipelotrichaceae*.

The family *Erysipelotrichaceae* was created in 2004 [27] and includes the 10 following genera: *Allobaculum* [28], *Bulleidia* [29], *Catenibacterium* [30], *Coprobacillus* (Kageyama and Benno 2000) [30], *Eggerthia* [31], *Erysipelothrix* [32], *Holdemania* [33], *Kandleria* [31], *Solobacterium* [30] and *Turicibacter* [34]. Currently, 12 species with validly published names are reported in this family [35]. The species listed in the *Erysipelotrichaceae* are mostly comprised of Gram-positive, non-spore forming, rod-shaped, straight or slightly curved or irregularly shaped, facultatively anaerobic or anaerobic, catalase negative, chemoorganotrophic fermentative or respiratory metabolism, acidifying glucose and other sugars [35]. Members of species within the radiation of *Erysipelotrichaceae* were identified as pathogens in both humans and animals. In humans, these bacteria were isolated from patients with oral infection and acute appendicitis [36-40].

## Classification and features

A stool sample was collected from a healthy 16-year-old male Senegalese volunteer patient living in Dielmo (rural village in the Guinean-Sudanian zone in Senegal), who was included in a research protocol. Written assent was obtained from this individual. For this study, no written consent was needed from his guardians because he was older than 15 years (in accordance with the previous project approved by the Ministry of Health of Senegal and the assembled village population and as published elsewhere [41].) Both this study and the assent procedure were approved by the National Ethics Committee of Senegal (CNERS) and the Ethics Committee of the Institut Fédératif de Recherche IFR48, Faculty of Medicine, Marseille, France (agreement numbers 09-022 and 11-017 Several other new bacterial species were isolated from this specimen using various culture conditions, including the recently described *Alistipes senegalensis*, *Alistipes timonensis*, *Anaerococcus senegalensis*,*Clostridium senegalense*, *Peptoniphilus timonensis*, *Paenibacillus senegalensis*, *Herbaspirillum massiliense*, *Kurthia massiliensis*, *Brevibacterium senegalense*, *Aeromicrobium massilense*, *Cellulomonas massiliensis*, *Senegalemassilia anaerobia*, *Peptoniphilus senegalensis* and *Enterobacter massiliensis* [9-20,23].

The fecal specimen was preserved at -80°C after collection. Strain JC13 ^T^ (Table 1) was isolated in January 2011 by cultivation on Brain Heart Infusion agar (Becton Dickinson, Pont de Claix, France), after a 10 day preincubation in anaerobic blood culture bottle.

**Table 1:** Classification and general features of *Dielma fastidiosa* strain JC13^T^

**MIGS ID**	**Property**	**Term**	**Evidence code^a^**
		Domain *Bacteria*	TAS [42]
		Phylum *Firmicutes*	TAS [43-45]
		Class *Erysipelotrichia*	TAS [46,47]
	Current classification	Order *Erysipelotrichales*	TAS [47,48]
		Family *Erysipelotrichaceae*	TAS [27]
		Genus *Dielma*	IDA
		Species *Dielma fastidiosa*	IDA
		Type strain JC13 ^T^	IDA
	Gram stain	Negative	IDA
	Cell shape	Rod	IDA
	Motility	Motile	IDA
	Sporulation	Non-sporulating	IDA
	Temperature range	Mesophile	IDA
	Optimum temperature	37°C	IDA
MIGS-6.3	Salinity	Unknown	IDA
MIGS-22	Oxygen requirement	Anaerobic	IDA
	Carbon source	Unknown	
	Energy source	Unknown	
MIGS-6	Habitat	Human gut	IDA
MIGS-15	Biotic relationship	Free living	IDA
MIGS-14	Pathogenicity Biosafety level Isolation	Unknown 2 Human feces	
MIGS-4	Geographic location	Senegal	IDA
MIGS-5	Sample collection time	September 2010	IDA
MIGS-4.1	Latitude	13.7167	IDA
MIGS-4.1	Longitude	-16.4167	IDA
MIGS-4.3	Depth	Surface	IDA
MIGS-4.4	Altitude	51 m above sea level	IDA

Evidence codes - IDA: Inferred from Direct Assay; TAS: Traceable Author Statement (i.e., a direct report exists in the literature); NAS: Non-traceable Author Statement (i.e., not directly observed for the living, isolated sample, but based on a generally accepted property for the species, or anecdotal evidence). These evidence codes are from the Gene Ontology project [49]. If the evidence is IDA, then the property was directly observed for a live isolate by one of the authors or an expert mentioned in the acknowledgements.

**Figure 1:**
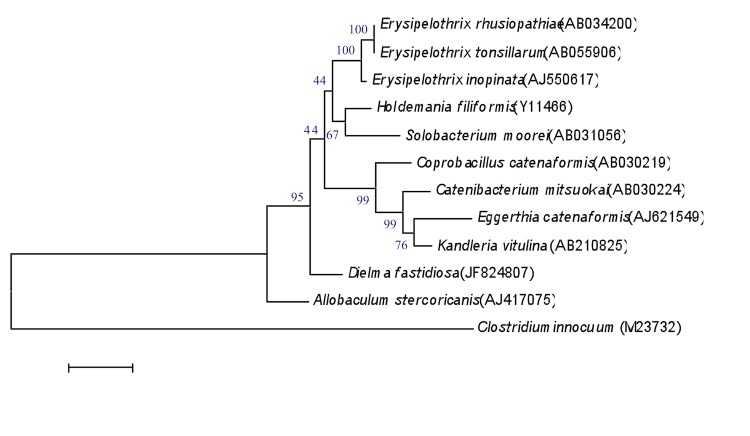
Phylogenetic tree highlighting the position of *D. fastidiosa* strain JC113^T^ relative to other type strains within the *Erysipelotrichaceae* family*.* GenBank accession numbers are indicated in parentheses. Sequences were aligned using CLUSTALW, and phylogenetic inferences obtained using the maximum-likelihood method within the MEGA software. Numbers at the nodes are percentages of bootstrap values obtained by repeating the analysis 500 times to generate a majority consensus tree. *C. innocuum* was used as outgroup. The scale bar represents a 1% nucleotide sequence divergence.

The 16S rRNA sequence (GenBank accession number JF824807) of *D. fastidiosa* strain JC13^T^ was compared to sequences in GenBank using BLAST [50] and showed a highest similarity of 89.71% with *Clostridium innocuum* (Figure 1). By comparison with type species from genera within the family *Erysipelotrichaceae*, *D. fastidiosa* exhibited a 16S rRNA sequence similarity ranging from 69.90 to 89.71%. Since these values are lower than the 95% threshold recommended by Stackebrandt and Ebers to delineate new genera without performing DDH [3], we propose to classify strain JC13^T^ within a novel genus.

**Figure 2:**
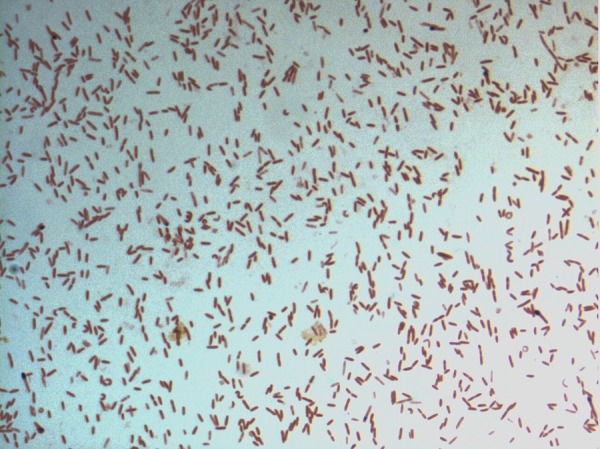
Gram staining of *D. fastidiosa* strain JC13^T^

**Figure 3:**
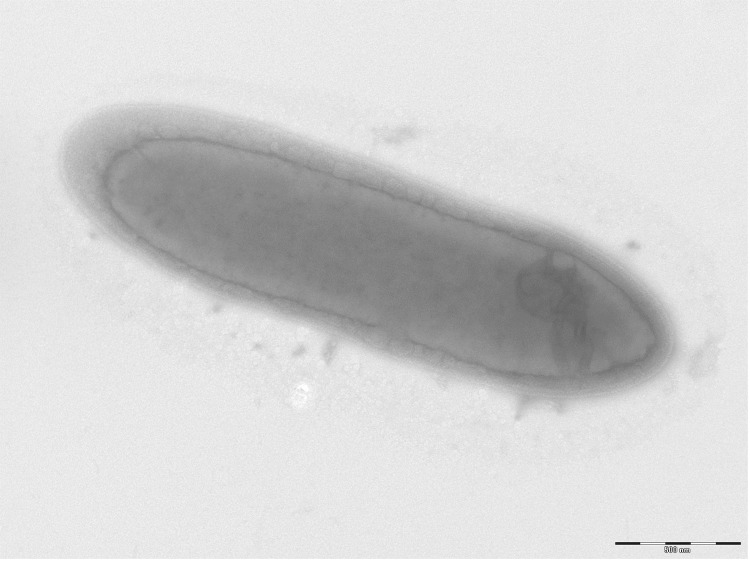
Transmission electron microscopy of *D. fastidiosa* strain JC13^T^, using a Morgani 268D (Philips) at an operating voltage of 60kV. The scale bar represents 500 nm.

Different growth temperatures (25, 30, 37, 45°C) were tested; growth occurred between 25°C and 45°C and optimal growth was observed at 30°C. Colonies were 0.5 to 1 mm in diameter on blood-enriched Columbia agar and BHI agar. Growth of the strain was tested under anaerobic and microaerophilic conditions using GENbag anaer and GENbag microaer systems, respectively (BioMérieux) and in the presence of air with or without 5% CO_2_. Growth was achieved only under anaerobic conditions. Gram staining showed a rod-shaped Gram-negative bacterium (Figure 2). The motility test was positive. Cells grown on agar have a mean diameter of 0.60 µm and a mean length of 2.2 µm in electron microscopy (Figure 3).

Strain JC13^T^ did not exhibit catalase or oxidase activity. Using API Rapid ID 32A, positive reactions were obtained for α-fucosidase and pyroglutamic acid arylamidase. Negative reactions were observed for indole production, nitrate reduction, urease, arginine dihydrolase, α-galactosidase, β-galactosidase 6 phosphate, α-glucosidase, β-glucosidase, α-arabinosidase, β-glucuronidase, N-acetyl-β-glucosaminidase, mannose and raffinose fermentation, glutamic acid decarboxylase, alkanine phospatase, arginine arylamidase, proline arylamidase, leucyl glycine arylamidase, phenylalanine aylamidase, leucine arylamidase, pyroglutamic acid arylamidase, tyrosine arylamidase, alanine arylamidase, glycine arylamidase, histidine arylamidase, glutamyl glutamic acid arylamidase, and serine arylamidase. Using an API 20NE strip, a positive reaction was observed for esculine hydrolysis. No sugar fermentation was observed using API 50CH (Biomerieux). *D. fastidiosa is* susceptible to amoxicillin, imipenem, metronidazole and ciprofloxacine, but resistant to trimethoprim/sulfamethoxazole, rifampin, doxycycline, and gentamicin. The differential phenotypic characteristics with other species are summarized in Table 2.

**Table 2:** Differential characteristics of *Dielma fastidiosa* strain JC13^T^, *Erysipelothrix inopinata* strain MF-EP02, *Holdemania filiormis* strain ATCC51649^T^, *Eggerthia catenaformis* strain JCM1121^T^, *Kandleria vitulina* strain JCM1143^T^

**Properties**	*D. fastidiosa*	*E. inopinata*	*H. filiformis*	*E. catenaformis*	*K. vitulina*
Cell diameter (µm)	0.60	0.5	na	na	na
Oxygen requirement	anaerobic	facultative anaerobic	anaerobic	anaerobic	anaerobic
Gram stain	–	+	+	+	+
Motility	+	–		–	–
**Production of**					
Acid arylamidase	+	+	na	na	na
Catalase	–	–	–	–	–
Oxidase	–	–	na	na	na
Nitrate reductase	–	na	–	na	na
Urease	–	na	na	na	na
β-galactosidase	–	na	na	na	na
N-acetyl-glucosamine	–	+	na	na	na
**Acid from**					
L-Arabinose	–	–	–	–	–
Ribose	–	w	–	–	–
Mannose	–	–	–	+	+
Mannitol	–	–	–	–	–
Sucrose	–	–	+	+	+
D-glucose	–	na	+	+	+
D-fructose	–	na	+	+	+
D-maltose	–	–	w	–	+
D-lactose	–	–	w	+	+
**Hydrolysis of**					
Esculin	+	na	+	+	+
**G+C content (mol%)**	40.0	37.5	na	34.8	34.4
**Habitat**	human gut	Vegetable broth	Human gut	Human gut	Bovine rumen

**Figure 4:**
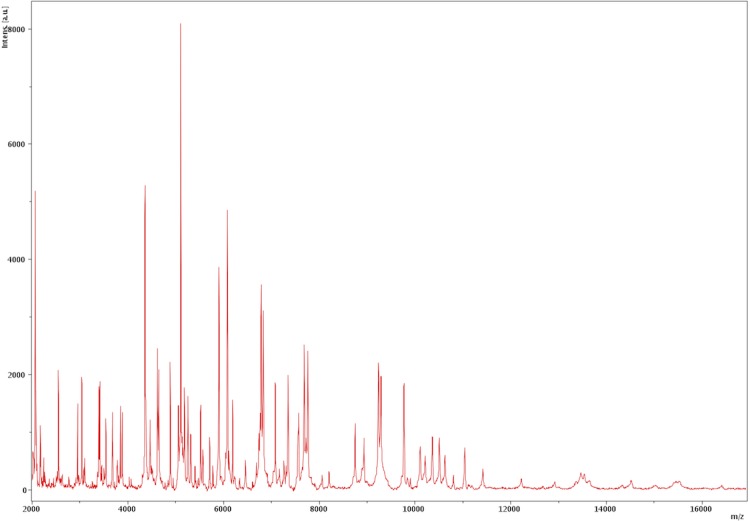
Reference mass spectrum from *D. fastidiosa* strain JC13^T^. Spectra from 12 individual colonies were compared and a reference spectrum was generated.

**Figure 5:**
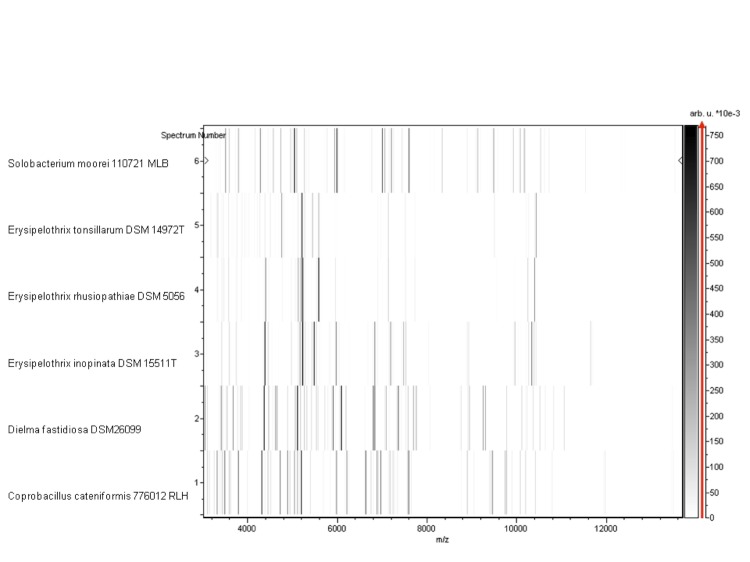
Gel view comparing *D. fastidiosa* gen. nov. sp. nov strain JC13^T^ and other species within *Erysipelotrichaceae* family. The gel view displays the raw spectra of loaded spectrum files arranged in a pseudo-gel like look. The x-axis records the m/z value. The left y-axis displays the running spectrum number originating from subsequent spectra loading. The peak intensity is expressed by a Gray scale scheme code. The color bar and the right y-axis indicate the relation between the color a peak is displayed with and the peak intensity in arbitrary units. Displayed species are indicated on the left.

Matrix-assisted laser-desorption/ionization time-of-flight (MALDI-TOF) MS protein analysis was carried out as previously described using a Microflex spectrometer (Bruker Daltonics, Leipzig, Germany) [7,51]. Briefly, a pipette tip was used to pick one isolated bacterial colony from a cultured agar plate, and spread it as a thin film on a MTP 384 MALDI-TOF target plate (Bruker Daltonics). Twelve distinct deposits were made for strain JC13 from twelve isolated colonies. Each smear was overlaid with 2µL of matrix solution (saturated solution of alpha-cyano-4-hydroxycinnamic acid) in 50% acetonitrile, 2.5% tri-fluoracetic-acid, and allowed to dry for five minutes. Measurements were performed with a Microflex spectrometer (Bruker). Spectra were recorded in the positive linear mode for the mass range of 2,000 to 20,000 Da (parameter settings: ion source 1 (IS1), 20 kV; IS2, 18.5 kV; lens, 7 kV). A spectrum was obtained after 675 shots at a variable laser power. The time of acquisition was between 30 seconds and 1 minute per spot. The twelve JC13 spectra were imported into the MALDI BioTyper software (version 2.0, Bruker) and analyzed by standard pattern matching (with default parameter settings) against the reference spectra from 4,334 bacteria (as updated on August 29, 2012), including spectra from 17 species within the *Erysipelotrichaceae*, contained in the BioTyper database. The method of identification included the m/z from 3,000 to 15,000 Da. For every spectrum, a maximum of 100 peaks were taken into account and compared with spectra in the database. A score enabled the identification, or not, from the tested species: a score > 2 with a validly published species enabled the identification at the species level, a score > 1.7 but < 2 enabled the identification only at the genus level; and a score < 1.7 did not enable any identification. For strain JC13^T^, no significant score was obtained, suggesting that our isolate was not a member of any known species or genus in the Biotyper database. We incremented our database with the spectrum from strain JC13^T^ (Figure 4). The gel view allowed us to highlight the spectra differences with other species of *Erysipelothrichaceae* family members (Figure 5).

## Genome sequencing information

### Genome project history

The organism was selected for sequencing on the basis of its phylogenetic position and 16S rRNA similarity to members of the family *Erysipelotrichaceae* and is part of a study of the human digestive flora aiming at isolating all bacterial species within human feces [1]. It was the seventh genome from the *Erysipelotrichaceae* family and the first genome of *Dielma fastidiosa* gen. nov., sp. nov. The Genbank accession number is CAEN00000000 and consists of 82 contigs. Table 3 shows the project information and its association with MIGS version 2.0 compliance [52]

**Table 3:** Project information

**MIGS ID**	**Property**	**Term**
MIGS-31	Finishing quality	High-quality draft
MIGS-28	Libraries used	454 GS paired-end 3- kb libraries
MIGS-29	Sequencing platform	454 GS FLX Titanium
MIGS-31.2	Sequencing coverage	28.92×
MIGS-30	Assemblers	gsAssembler
MIGS-32	Gene calling method	PRODIGAL
	Genbank Date of Release	December 19, 2012
	NCBI project ID	CAEN00000000
MIGS-13	Project relevance	Study of the human gut microbiome

### Growth conditions and DNA isolation

*Dielma fastidiosa* sp. nov., strain JC13^T^ (= CSUR P149 = DSM 26099) was grown on 5% sheep blood-enriched Columbia agar at 30°C in anaerobic atmosphere. Seven petri dishes were spread and the cultivated bacteria were resuspended in 3×100µl of G2 buffer (EZ1 DNA Tissue kit, Qiagen). A first mechanical lysis was performed with glass powder on a Fastprep-24 device (MP Biomedicals, USA) using 2×20 seconds cycles. DNA was then treated with 2.5µg/µL lysozyme for 30 minutes at 37°C and extracted through the BioRobot EZ1 Advanced XL (Qiagen). The DNA was then concentrated and purified with a QIAamp kit (Qiagen). The yield and concentration was measured using a Quant-it Picogreen kit (Invitrogen) on a GeniosTecan fluorometer at 46.6ng/µl.

### Genome sequencing and assembly

DNA (5µg) was mechanically fragmented with a Hydroshear device (Digilab, Holliston, MA, USA) with an enrichment size at 3-4kb. The DNA fragmentation was visualized through the Agilent 2100 BioAnalyzer on a DNA labchip 7500 with an optimal size of 3.4kb. The library was constructed according to the 454 GS FLX Titanium paired-end protocol (Roche). Circularization and nebulization were performed. After PCR amplification through 15 cycles followed by double size selection, the single stranded paired-end library profile was visualized on an Agilent 2100 RNA Pico 6000 Labchip with an optimal length of 522 bp. Then the library was quantified on the Quant-it Ribogreen kit (Invitrogen) on a GeniosTecan fluorometer at 133 pg/µL. The library concentration equivalence was calculated as 4.67E+08 molecules/µL. The library was stored at -20°C until further use.

The shotgun library was clonally amplified with 0.5cpb in 4 emPCR reactions and 1cpb in 4 emPCR reactions with the GS Titanium SV emPCR Kit (Lib-L) v2 (Roche). The yields of the emPCRs were 5.10% and 10.73%, respectively, in the range of 5 to 20% recommended by the Roche procedure. Twice, approximately 790,000 beads were loaded on a GS Titanium PicoTiterPlate PTP Kit 70×75 and sequenced with the GS Titanium Sequencing Kit XLR70 (Roche). The runs were performed overnight and analyzed on the cluster through the gsRunBrowser and Newbler assembler (Roche). A total of 428,372 passed filter wells were obtained and generated 101.3Mb of sequences with an average of length of 219 bp. The passed filter sequences were assembled using Newbler (Roche) with 90% identity and 40 bp as overlap. The final assembly identified 22 scaffolds and 82 large contigs (>1,500bp), and generated a genome size of 3.57Mb which corresponds to a coverage of 28.92x genome equivalent.

### Genome annotation

Open reading frames (ORFs) were predicted using Prodigal [53] with default parameters. However, the predicted ORFs were excluded if they spanned a sequencing gap region. The predicted bacterial protein sequences were searched against the GenBank [54] and Clusters of Orthologous Groups (COG) databases using BLASTP. The tRNAs and rRNAs were predicted using the tRNAScanSE [55] and RNAmmer [56] tools, respectively. Lipoprotein signal peptides and numbers of transmembrane helices were predicted using SignalP [57] and TMHMM [58], respectively. ORFans were identified if their BLASTP *E*-value was lower than 1e-03 for alignment length greater than 80 amino acids. If alignment lengths were smaller than 80 amino acids, we use an *E*-value of 1e-05. Such parameter thresholds have already been used in previous works to define ORFans. Artemis [59] and DNA Plotter [60] were used for data management and visualization of genomic features, respectively. The Mauve alignment tool (version 2.3.1) was used for multiple genomic sequence alignment [61]. To estimate the mean level of nucleotide sequence similarity at the genome level between *D. fastidiosa* JC13^T^ and another 6 genomes from members of the *Erysipelotrichaceae* family (Table 4), orthologous proteins were detected using the Proteinortho [62] and we compared genomes two by two and determined the mean percentage of nucleotide sequence identity among orthologous ORFs using BLASTn.

**Table 4:** Genomic comparison of *D. fastidiosa* JC13^T^ with six other members of *Erysipelotrichaceae* family

Species	Strain	Genome accession number	Genome Size (Mb)	G+C content %
*Dielma fastidiosa*	JC13^T^	CAEN00000000	3,575,363	40.00
*Erysipelothrix rhusiopathiae*	Fujisawa	NC_015601	1,787,941	36.60
*Holdemania filiformis*	DSM 12042	NZ_ACCF00000000	3,803,745	50.20
*Bulleidia extructa*	W1219	NZ_ADFR00000000	3,170963	36.20
*Catenibacterium mitsuokai*	DSM 15897	NZ_ACCK00000000	4,835437	36.80
*Solobacterium moorei*	F0204	NZ_AECQ00000000	4,403,767	36.80
*Turicibacter sanguinis*	PC909	NZ_ADMN00000000	2,953,411	34.10

## Genome properties

**Figure 6:**
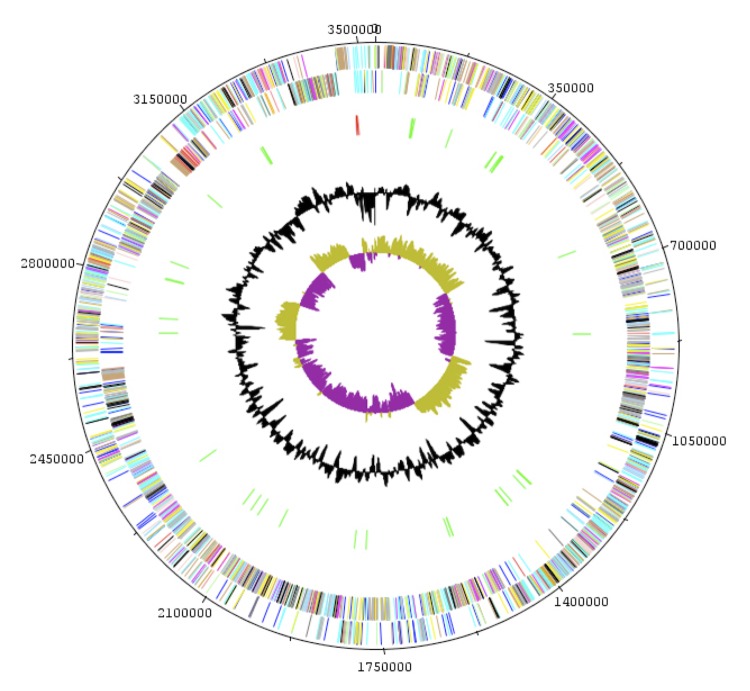
Graphical circular map of the chromosome. From outside to the center: Genes on the forward strand (colored by COG categories), genes on the reverse strand (colored by COG categories), RNA genes (tRNAs green, rRNAs red), GC content, and GC skew.

The genome is 3,574,031 bp long (one chromosome of 3,556,241 bp and one plasmid of 17,790 bp) with a GC content of 40.00% (Figure 6 and Table 5). Of the 3,491 predicted chromosomal genes, 3,441 were protein-coding genes and 50 were RNAs. A total of 2,534 genes (72.58%) were assigned a putative function. ORFans accounted for 269 genes (7.81%) and the remaining genes were annotated as hypothetical proteins. The properties and statistics of the genome are summarized in Tables 5 and 6. The distribution of genes into COGs functional categories is presented in Table 6. The 17,790bp-long plasmid contains 17 protein-coding genes. A BLASTN search showed its closest match to be the DO plasmid from *Enterococcus faecium* (GenBank Accession number: NC017961).

**Table 5:** Nucleotide content and gene count levels of the chromosome

**Attribute**	Value	% of total^a^
Genome size (bp)	3,556,241	
DNA coding region (bp)	3,170,580	89.15
DNA G+C content (bp)	1,422,496	40.0
Total genes	3,491	100
RNA genes	50	1.43
Protein-coding genes	3,441	98.56
Genes with function prediction	2,534	72.58
Genes assigned to COGs	2,329	66.71
Genes with peptide signals	259	7.41
Genes with transmembrane helices	905	25.62

^a^ The total is based on either the size of the genome in base pairs or the total number of protein coding genes in the annotated genome

**Table 6:** Number of genes associated with the 25 general COG functional categories

**Code**	**Value**	**%age**^a^	**Description**
J	149	4.33	Translation
A	1	0.029	RNA processing and modification
K	163	4.73	Transcription
L	176	5.11	Replication, recombination and repair
B	0	0	Chromatin structure and dynamics
D	24	0.69	Cell cycle control, mitosis and meiosis
Y	0	0	Nuclear structure
V	47	1.36	Defense mechanisms
T	52	1.511	Signal transduction mechanisms
M	105	3.05	Cell wall/membrane biogenesis
N	0	0	Cell motility
Z	0	0	Cytoskeleton
W	0	0	Extracellular structures
U	26	0.75	Intracellular trafficking and secretion
O	76	2.20	Posttranslational modification, protein turnover, chaperones
C	139	4.03	Energy production and conversion
G	88	2.55	Carbohydrate transport and metabolism
E	189	5.49	Amino acid transport and metabolism
F	63	1.83	Nucleotide transport and metabolism
H	72	2.09	Coenzyme transport and metabolism
I	123	3.57	Lipid transport and metabolism
P	127	3.69	Inorganic ion transport and metabolism
Q	25	0.72	Secondary metabolites biosynthesis, transport and catabolism
R	250	7.26	General function prediction only
S	182	5.28	Function unknown
-	301	8.74	Not in COGs

^a^ The total is based on the total number of protein coding genes in the annotated genome

### Genome comparison of *D. fastidiosa* with other genomes of *Erysipelotrichaceae* family

Here, we compared the genome of *D. fastidiosa* JC13T with 6 other genomes from *Erysipelotrichaceae* family (Table 3 and 6). *D. fastidiosa* (3.57 Mb) is larger than those of *E. rhusiopathiae*, *B. extructa* and *T. sanguinis* (1.78, 3.17 and 2.95 Mb respectively) but smaller than those of *H. filiformis*, *C. mitsuokai* and *S. moorei* (3.80, 4.83 and 4.40 Mb respectively). The G+C content of *D. fastidiosa* is higher (40.0%) than all the genomes compared except *H. filiformis* (50.2%). *D. fastidiosa* has more predicted genes than *S. moorei* and *T. sanguinis* (2,619, 2,342 and 2,335 respectively) but less than those of *E. rhusiopathiae*, *H. filiformis*, *B. extructa* and *C. mitsuokai* (2,709, 3,161, 3,132 and 3,231, respectively). In addition, *D. fastidiosa* shared 681, 1,005, 650, 804, 824 and 781 orthologous genes with *E. rhusiopathiae*, *H. filiformis*, *B. extructa*, *C. mitsuokai*, *S. moorei* and *T. sanguinis* respectively. The average nucleotide sequence identity ranged from 61.12 to 69.65% among *Erysipelotrichaceae* family species, and from 64.29 to 65.99% between *D. fastidiosa* and other species, thus confirming its new species status (Table 7).

**Table 7:** The numbers of orthologous protein shared between genomes (above diagonal), average percentage similarity of nucleotides corresponding to orthologous protein shared between genomes (below diagonal) and the numbers of proteins per genome (bold).

Species	*D. fastidiosa*	*E. rhusiopathiae*	*H. filiformis*	*B. extructa*	*C. mitsuokai*	*S. moorei*	*T. sanguinis*
*Dielma fastidiosa*	**2,619**	681	1,005	650	804	824	*781*
*Erysipelothrix rhusiopathiae*	64.99	**2,709**	650	534	553	597	*563*
*Holdemania filiformis*	65.99	62.24	**3,161**	661	761	808	*749*
*Bulleidia extructa*	64.89	65.24	62.84	**3,132**	539	745	*524*
*Catenibacterium mitsuokai*	65.13	65.13	61.12	65.13	**3,231**	649	*659*
*Solobacterium moorei*	65.16	65.54	63.13	69.65	66.18	**2,342**	614
*Turicibacter sanguinis*	64.29	65.37	64.62	64.76	65.89	64.62	**2,335**

## Conclusion

On the basis of phenotypic, phylogenetic and genome analysis, we formally propose the creation of *Dielma fastidiosa gen nov.,* sp. nov., that contains strain JC13^T^. This strain has been found in Senegal.

### Description of Dielma *gen. nov.*

*Dielma* (di.el’ma, N.L. fem. N. Dielma, of Dielmo, the Senegalese village where lived the patient from whom strain JC13^T^ was cultivated).

Gram-negative rods. Strictly anaerobic. Mesophilic. Motile. Negative for catalase, oxidase, nitrate reduction and indole production. Positive for α-fucosidase, pyroglutamic acid arylamidase and esculine hydrolysis. Habitat: human digestive tract. Type species: *Dielma fastidiosa*.

### Description of *Dielma fastidiosa* sp. nov., gen nov.

*Dielma fastidiosa* (fas.ti.di.o’sa. N. L. F. adj. from the Latin adjective *fastidiosus* excessively sensitive; referring to the difficulty to isolate this microorganism). It has been isolated from feces from an asymptomatic Senegalese patient.

*Dielma fastidiosa* is an anaerobic Gram-negative bacterium. Growth is achieved only anaerobically. Growth occurs on axenic medium between 25 and 45°C, with optimal growth observed at 30°C. Cells stain Gram-negative, are rod-shaped, non-sporulating, motile and have a mean diameter of 0.60 µm and a mean length of 2.2 µm. Colonies are 0.5 to 1 mm in diameter on blood-enriched Columbia agar. Oxidase negative. Catalase negative. Using the API Rapid ID 32A system, positive reactions are obtained for α-fucosidase and pyroglutamic acid arylamidase. Negative reactions for indole production, nitrate reduction, urease, arginine dihydrolase, α-galactosidase, β-galactosidase 6 phosphate, α-glucosidase, β-glucosidase, α-arabinosidase, β-glucuronidase, N-acetyl-β-glucosaminidase, mannose and raffinose fermentation, glutamic acid decarboxylase, alkanine phospatase, arginine arylamidase, proline arylamidase, leucyl glycine arylamidase, phenylalanine aylamidase, leucine arylamidase, pyroglutamic acid arylamidase, tyrosine arylamidase, alanine arylamidase, glycine arylamidase, histidine arylamidase, glutamyl glutamic acid arylamidase, and serine arylamidase. With the API 20NE system, a positive reaction is observed for aesculin. Asaccharolytic. *D. fastidiosa* is susceptible to amoxicillin, imipenem, metronidazole and ciprofloxacin, but resistant to trimethoprim/sulfamethoxazole, rifampin, doxycycline and gentamicin.

The G+C content of the genome is 40%. The 16S rRNA and genome sequences are deposited in Genbank and EMBL under accession numbers JF824807 and CAEN00000000, respectively. The type strain is JC13^T^ (= CSUR P149 = DSM 26099) was isolated from the fecal flora of a healthy Senegalese patient.

## References

[1] Lagier JC Armougom F Million M Hugon P Pagnier I Robert C Bittar F Fournous G Gimenez G Maraninchi M Microbial culturomics: paradigm shift in the human gut microbiome study. Clin Microbiol Infect 2012; 18:1185-1193 23033984 10.1111/1469-0691.12023

[2] Dubourg G Lagier JC Armougom F Robert C Hamad I Brouqui P The gut microbiota of a patient with resistant tuberculosis is more comprehensively studied by culturomics than by metagenomics. Eur J Clin Microbiol Infect Dis 2013; 32:637-645 10.1007/s10096-012-1787-3 23291779

[3] Tindall BJ Rosselló-Móra R Busse HJ Ludwig W Kämpfer P Notes on the characterization of prokaryote strains for taxonomic purposes. Int J Syst Evol Microbiol 2010; 60:249-266 10.1099/ijs.0.016949-0 19700448

[4] Stackebrandt E Ebers J Taxonomic parameters revisited: tarnished gold standards. Microbiol Today 2006; 33:152-155

[5] Wayne LG Brenner DJ Colwell RR Grimont AD Kandler O Krichevsky MI Moore LH Moore EC Murray GE Sktackbrandt E Report of the ad hoc committee on reconciliation of approaches to bacterial systematic. Int J Syst Bacteriol 1987; 37:463-464 10.1099/00207713-37-4-463

[6] Rossello-Mora R. DNA-DNA Reassociation Methods Applied to Microbial Taxonomy and Their Critical Evaluation. In: Stackebrandt E (ed), Molecular Identification, Systematics, and population Structure of Prokaryotes. Springer, Berlin, 2006, p. 23-50.

[7] Welker M Moore ER Applications of whole-cell matrix-assisted laser-desorption/ionization time-of-flight mass spectrometry in systematic microbiology. Syst Appl Microbiol 2011; 34:2-11 10.1016/j.syapm.2010.11.013 21288677

[8] Kokcha S Mishra AK Lagier JC Million M Leroy Q Raoult D Fournier PE Non contiguous-finished genome sequence and description of *Bacillus timonensis* sp. nov. Stand Genomic Sci 2012; 6:346-355 10.4056/sigs.2776064 23408487 PMC3558959

[9] Lagier JC El Karkouri K Nguyen TT Armougom F Raoult D Fournier PE Non-contiguous finished genome sequence and description of *Anaerococcus senegalensis* sp. nov. Stand Genomic Sci 2012; 6:116-125 10.4056/sigs.2415480 22675604 PMC3359877

[10] Mishra AK Gimenez G Lagier JC Robert C Raoult D Fournier PE Non-contiguous finished genome sequence and description of *Alistipes senegalensis* sp. nov. Stand Genomic Sci 2012; 6:304-314 10.4056/sigs.2625821 PMC3569391 23407294

[11] Lagier JC Armougom F Mishra AK Ngyuen TT Raoult D Fournier PE Non-contiguous finished genome sequence and description of *Alistipes timonensis* sp. nov. Stand Genomic Sci 2012; 6:315-324 23408657 10.4056/sigs.2685971 PMC3558960

[12] Mishra AK Lagier JC Robert C Raoult D Fournier PE Non-contiguous finished genome sequence and description of *Clostridium senegalense* sp. nov. Stand Genomic Sci 2012; 6:386-395 23408737 10.4056/sigs.2766062 PMC3558962

[13] Mishra AK Lagier JC Robert C Raoult D Fournier PE Non contiguous-finished genome sequence and description of *Peptoniphilus timonensis* sp. nov. Stand Genomic Sci 2012; 7:1-11 10.4056/sigs.2956294 23449949 PMC3570796

[14] Mishra AK Lagier JC Rivet R Raoult D Fournier PE Non contiguous-finished genome sequence and description of *Paenibacillus senegalensis* sp. nov. [epub.]. Stand Genomic Sci 2012; 7:70-81 23459006 10.4056/sigs.3056450 PMC3577113

[15] Lagier JC Gimenez G Robert C Raoult D Fournier PE Non-contiguous finished genome sequence and description of *Herbaspirillum massiliense* sp. nov. Stand Genomic Sci 2012; 7:200-209 23407294 10.4056/sigs.3086474 PMC3569391

[16] Roux V El Karkouri K Lagier JC Robert C Raoult D Non-contiguous finished genome sequence and description of *Kurthia massiliensis* sp. nov. Stand Genomic Sci 2012; 7:221-232 10.4056/sigs.3206554 23407462 PMC3569394

[17] Kokcha S Ramasamy D Lagier JC Robert C Raoult D Fournier PE Non-contiguous finished genome sequence and description of *Brevibacterium senegalense* sp. nov. Stand Genomic Sci 2012; 7:233-245 10.4056/sigs.3256677 23408786 PMC3569389

[18] Ramasamy D Kokcha S Lagier JC N’Guyen TT Raoult D Fournier PE Non-contiguous finished genome sequence and description of *Aeromicrobium massilense* sp. nov. Stand Genomic Sci 2012; 7:246-257 10.4056/sigs.3306717 23408786 PMC3569389

[19] Lagier JC Ramasamy D Rivet R Raoult D Fournier PE Non-contiguous finished genome sequence and description of *Cellulomonas massiliensis* sp. nov. Stand Genomic Sci 2012; 7:258-270 10.4056/sigs.3316719 23408774 PMC3569388

[20] Lagier JC El Karkouri K Rivet R Couderc C Raoult D Fournier PE Non-contiguous finished genome sequence and description of *Senegalemassilia anaerobia* sp. nov. Stand Genomic Sci 2013; 7:343-356 10.4056/sigs.3246665 PMC3764928 24019984

[21] Mishra AK Hugon P Lagier JC Nguyen TT Robert C Couderc C Raoult D Fournier PE Non-contiguous finished genome sequence and description of *Peptoniphilus obesi* sp. nov. Stand Genomic Sci 2013; 7:357-369 10.4056/sigs.32766871 PMC3764929 24019985

[22] Mishra AK Lagier JC Nguyen TT Raoult D Fournier PE Non-contiguous finished genome sequence and description of *Peptoniphilus senegalensis* sp. nov. Stand Genomic Sci 2013; 7:370-381 10.4056/sigs.3366764 PMC3764932 24019986

[23] Lagier JC El Karkouri K Mishra AK Robert C Raoult D Fournier PE Non-contiguous finished genome sequence and description of *Enterobacter massiliensis* sp. nov. Stand Genomic Sci 2013; 7:399-412 10.4056/sigs.3396830 PMC3764934 24019988

[24] Hugon P Ramasamy D Lagier JC Rivet R Couderc C Raoult D Fournier PE Non-contiguous finished genome sequence and description of *Alistipes obesi* sp. nov. Stand Genomic Sci 2013; 7:427-439 10.4056/sigs.3336746 PMC3764931 24019990

[25] Mishra AK Hugon P Robert C Couderc C Raoult D Fournier PE Non-contiguous finished genome sequence and description of *Peptoniphilus grossensis* sp. nov. Stand Genomic Sci 2012; 7:320-330 23408485 10.4056/sigs.3076460 PMC3569384

[26] Hugon P Mishra AK Lagier JC Nguyen TT Couderc C Raoult D Fournier PE Non-contiguous finished genome sequence and description of *Brevibacillus massiliensis* sp. nov. Stand Genomic Sci 2013; 8:1-14 10.4056/sigs.3466975 23961307 PMC3739172

[27] Verbarg S Rheims H Emus S Frühling A Kroppenstedt RM Stackebrandt E Schumann P *Erysipelothrix inopinata* sp. nov., isolated in the course of sterile filtration of vegetable peptone broth, and description of Erysipelotrichaceae fam. nov. Int J Syst Evol Microbiol 2004; 54:221-225 10.1099/ijs.0.02898-0 14742484

[28] Greetham HL Gibson GR Giffard C Hippe H Merkhoffer B Steiner U Falsen E Collins MD *Allobaculum stercoricanis* gen. nov., sp. nov., isolated from canine feces. Anaerobe 2004; 10:301-307 10.1016/j.anaerobe.2004.06.004 16701531

[29] Downes J Olsvik B Hiom SJ Spratt DA Cheeseman SL Olsen I Weightman AJ Wade WG *Bulleidia extructa* gen. nov., sp. nov., isolated from the oral cavity. Int J Syst Evol Microbiol 2000; 50:979-983 10.1099/00207713-50-3-979 10843035

[30] Kageyama A Benno Y *Catenibacterium mitsuokai* gen. nov., sp. nov., a gram-positive anaerobic bacterium isolated from human faeces. Int J Syst Evol Microbiol 2000; 50:1595-1599 10.1099/00207713-50-4-1595 10939666

[31] Salvetti E Felis GE Dellaglio F Castioni A Torriani S Lawson PA Reclassification of *Lactobacillus catenaformis* (Eggerth 1935) Moore and Holdeman 1970 and *Lactobacillus vitulinus* Sharpe et al. 1973 as *Eggerthia catenaformis* gen. nov., comb. nov. and *Kandleria vitulina* gen. nov., comb. nov., respectively. Int J Syst Evol Microbiol 2011; 61:2520-2524 10.1099/ijs.0.029231-0 21112984

[32] Skerman (V.B.D.). McGowan (V.) and Sneath (P.H.A.) (editors): Approved Lists of Bacterial Names. Int. J. Syst. Bacteriol., 1980, 30, 225-420.

[33] Willems A Moore WE Weiss N Collins MD Phenotypic and phylogenetic characterization of some *Eubacterium*-like isolates containing a novel type B wall murein from human feces: description of *Holdemania filiformis* gen. nov., sp. nov. Int J Syst Bacteriol 1997; 47:1201-1204 10.1099/00207713-47-4-1201 9336928

[34] Bosshard PP Zbinden R Altwegg M *Turicibacter sanguinis* gen. nov., sp. nov., a novel anaerobic, Gram-positive bacterium. Int J Syst Evol Microbiol 2002; 52:1263-1266 10.1099/ijs.0.02056-0 12148638

[35] List of Prokaryotic names with standing nomenclature (LPSN). http://www.bacterio.cict.fr

[36] Brooke CJ Riley TV *Erysipelothrix rhusiopathiae*: Bacteriology, epidemiology and clinical manifestations of an occupational pathogen. J Med Microbiol 1999; 48:789-799 10.1099/00222615-48-9-789 10482289

[37] Barber M Discussion on swine erysipelas infection (Erysipelothrix rhusiopathiae) in man and animals. Proc R Soc Med 1948; 41:328-332 18864715 10.1177/003591574804100543 PMC2184626

[38] Downes J Olsvik B Hiom SJ Spratt DA Cheeseman SL Olsen I Weightman AJ Wade WG *Bulleidia extructa* gen. nov., sp. nov., isolated from the oral cavity. Int J Syst Evol Microbiol 2000; 50:979-983 10.1099/00207713-50-3-979 10843035

[39] Takahashi T Fujisawa T Benno Y Tamura Y Sawada T Suzuki S Muramatsu M Mitsuoka T *Erysipelothrix tonsillarum* sp. nov. isolated from tonsils of apparently healthy pigs. Int J Syst Bacteriol 1987; 37:166-168 10.1099/00207713-37-2-166

[40] Allianatos PG Tilentzoglou AC Koutsoukou AD Septic arthritis caused by *Erysipelothrix rhusiopathiae* infection after arthroscopically assisted anterior cruciate ligament reconstruction. Arthroscopy 2003; 19:26 10.1053/jars.2003.50077 12627143

[41] Trape JF Tall A Diagne N Ndiath O Ly AB Faye J Dieye-Ba F Roucher C Bouganali C Badiane A Malaria morbidity and pyrethroid resistance after the introduction of insecticide-treated bednets and artemisinin-based combination therapies: a longitudinal study. Lancet Infect Dis 2011; 11:925-932 10.1016/S1473-3099(11)70194-3 21856232

[42] Woese CR Kandler O Wheelis ML Towards a natural system of organisms: proposal for the domains *Archae, Bacteria*, and *Eukarya.* Proc Natl Acad Sci USA 1990; 87:4576-4579 10.1073/pnas.87.12.4576 2112744 PMC54159

[43] Murray RGE. The higher taxa, or, a place for everything...? *In*: Krieg NR, Holt JG (eds), *Bergey's Manual of Systematic Bacteriology*, First edition, volume 1, The Williams & Wilkins Co., Baltimore, 1984, p. 31-34.

[44] Gibbons NE Murray RGE Proposals concerning the higher taxa of *Bacteria.* Int J Syst Bacteriol 1978; 28:1-6 10.1099/00207713-28-1-1

[45] Garrity GM, Holt J. The road map to the manual. In: Garrity GM, Boone DR, Castenholz RW (eds), *Bergey's Manual of Systematic Bacteriology* Second Edition, Volume 1, Springer, New York, 2001, p.119-169.

[46] Ludwig W, Schleifer KH, Whitman WB. Class III. *Erysipelotrichia* class nov. In: De Vos P, Garrity G, Jones D, Krieg NR, Ludwig W, Rainey FA, Schleifer KH, Whitman WB (eds): Bergey's Manual of Systematic Bacteriology, second edition, vol. 3 (The *Firmicutes*), Springer, Dordrecht, Heidelberg, London, New York, 2009, p. 1298.

[47] List Editor List of new names and new combinations previously effectively, but not validly, published. List no. 132. Int J Syst Evol Microbiol 2010; 60:469-472 10.1099/ijs.0.022855-0 20458120

[48] Ludwig W, Schleifer KH, Whitman WB. Order I. *Erysipelotrichales* ord. nov. In: De Vos P, Garrity G, Jones D, Krieg NR, Ludwig W, Rainey FA, Schleifer KH, Whitman WB (eds), Bergey's Manual of Systematic Bacteriology, Second Edition, Volume 3, Springer-Verlag, New York, 2009, p. 1298.

[49] Ashburner M Ball CA Blake JA Botstein D Butler H Cherry JM Davis AP Dolinski K Dwight SS Eppig JT Gene ontology: tool for the unification of biology. The Gene Ontology Consortium. Nat Genet 2000; 25:25-29 10.1038/75556 10802651 PMC3037419

[50] Altschul SF Gish W Miller W Myers EW Lipman DJ Basic local alignment search tool. J Mol Biol 1990; 215:403-410 2231712 10.1016/S0022-2836(05)80360-2

[51] Seng P Drancourt M Gouriet F La Scola B Fournier PE Rolain JM Raoult D Ongoing revolution in bacteriology: routine identification of bacteria by matrix-assisted laser desorption ionization time-of-flight mass spectrometry. Clin Infect Dis 2009; 49:543-551 10.1086/600885 19583519

[52] Field D Garrity G Gray T Morrison N Selengut J Sterk P Tatusova T Thomson N Allen MJ Angiuoli SV The minimum information about a genome sequence (MIGS) specification. Nat Biotechnol 2008; 26:541-547 10.1038/nbt1360 18464787 PMC2409278

[53] Prodigal. http://prodigal.ornl.gov/

[54] Benson DA Karsch-Mizrachi I Clark K Lipman DJ Ostell J Sayers EW GenBank. Nucleic Acids Res 2012; 40:D48-D53 10.1093/nar/gkr1202 22144687 PMC3245039

[55] Lowe TM Eddy SR tRNAscan-SE: a program for improved detection of transfer RNA genes in genomic sequence. Nucleic Acids Res 1997; 25:955-964 9023104 10.1093/nar/25.5.955 PMC146525

[56] Lagesen K Hallin P Rodland EA Staerfeldt HH Rognes T Ussery DW RNAmmer: consistent and rapid annotation of ribosomal RNA genes. Nucleic Acids Res 2007; 35:3100-3108 10.1093/nar/gkm160 17452365 PMC1888812

[57] Bendtsen JD Nielsen H von Heijne G Brunak S Improved prediction of signal peptides: SignalP 3.0. J Mol Biol 2004; 340:783-795 10.1016/j.jmb.2004.05.028 15223320

[58] Krogh A Larsson B von Heijne G Sonnhammer EL Predicting transmembrane protein topology with a hidden Markov model: application to complete genomes. J Mol Biol 2001; 305:567-580 10.1006/jmbi.2000.4315 11152613

[59] Rutherford K Parkhill J Crook J Horsnell T Rice P Rajandream MA Barrell B Artemis: sequence visualization and annotation. Bioinformatics 2000; 16:944-945 10.1093/bioinformatics/16.10.944 11120685

[60] Carver T Thomson N Bleasby A Berriman M Parkhill J DNAPlotter: circular and linear interactive genome visualization. Bioinformatics 2009; 25:119-120 10.1093/bioinformatics/btn578 18990721 PMC2612626

[61] Darling AC Mau B Blattner FR Perna NT Mauve: multiple alignment of conserved genomic sequence with rearrangements. Genome Res 2004; 14:1394-1403 10.1101/gr.2289704 15231754 PMC442156

[62] Lechner M Findeib S Steiner L Marz M Stadler PF Prohaska SJ Proteinortho: Detection of (Co-) orthologs in large-scale analysis. BMC Bioinformatics 2011; 12:124 10.1186/1471-2105-12-124 21526987 PMC3114741

